# Reduction of radiation loss at small-radius bend using spoof surface plasmon polariton transmission line

**DOI:** 10.1038/srep41077

**Published:** 2017-01-23

**Authors:** Wen Xuan Tang, Hao Chi Zhang, Jun Feng Liu, Jie Xu, Tie Jun Cui

**Affiliations:** 1 State Key Laboratory of Millimetre Waves, School of Information Science and Engineering, Southeast University, Nanjing 210096, China

## Abstract

Spoof surface plasmon polariton (SPP) has been realized at low frequencies through corrugated metallic structures. As two-dimensional application, the ultrathin SPP transmission lines (TLs) have been proposed with great potentials for microwave compact circuits due to the strong field confinement and enhancement, as well as controllable dispersive properties. In this paper, we examine the radiation loss at small-radius bend, which may cause severe crosstalk in highly-integrated circuits or systems, for the SPP TLs. We theoretically analyze that the SPP TL has essential merit of low radiation loss, and show better performance of SPP TL than the conventional microstrip line through numerical simulations and experiments. Both simulated and measured results demonstrate that the new type of transmission line can efficiently suppress the radiation loss at small-radius bend, and hence reduce the crosstalk in circuits and systems.

Microwave communication may be one of the most popular applications of electromagnetic (EM) wave in the modern society. New physics and technology have been explored and developed continuously for integrated communication systems with low cost, limited size and high efficiency. One big challenge for large-scale high-integrated circuits is the crosstalk between planar transmission lines (TLs), especially when discontinuities such as bends or chamfers exist in TLs and, thereat, the radiation loss becomes significant. The “escaped” electromagnetic wave may depress the efficiency and accuracy of integrated system and even harm the electric circuits. Therefore, analysis and solutions on this issue have been intensively studied^1^,^2^,^3^.

Recently, artificial surface plasmon polariton (SPP) has been realized at microwave and THz frequency bands through corrugated metallic structures, termed as the spoof (or designer) SPP^4^,^5^,^6^. Electromagnetic energy is strongly confined in sub-wavelength-scaled unit cells and propagates in form of SPP wave, similar to what happens in nature at the interface between metal and dielectric in the optical regime. Advantages of spoof SPP such as high field confinement, low loss, and controllable dispersion properties can be utilized to build novel plasmonic waveguides and planar transmission lines (TLs)^7^,^8^. Such spoof SPP TL has been proved to surpass microstrip, one of the most popular microwave TLs, in terms of low-crosstalk and flexible EM property, and hence is considered as a promising candidate to break the challenge of signal integrity in compact-size and highly-integrated communication systems^9^.

In this paper, we further examine the EM property of spoof SPP TL in more complicated circuit when small-radius bend is included and radiation loss inevitably increases. We first analyze the physics behind radiation loss from curved open waveguide (SPP TL, microstrip line, *et al*.), as well as the solution to this issue. Next, we study the dispersion property for both SPP TL and microstrip line. In the end, we fabricate prototypes for both transmission lines, and demonstrate our theory numerically and experimentally.

## Results

### Radiation from curved open waveguide

**Figure 1:**
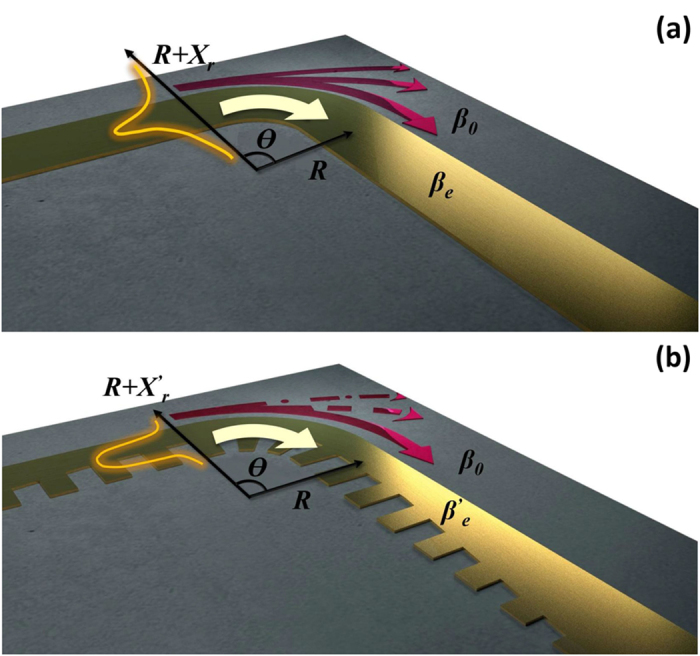
Generalized models of (**a**) a curved metallic transmission line and (**b**) an SPP transmission line with the same bend. Red arrows indicate how energy flows. A portion of energy is lost to radiation.

It has been analyzed that the radiation loss increases significantly at bends in open electromagnetic waveguides that have transverse field extending indefinitely into a freely propagating region (e.g. the dielectric waveguide)^10^,^11^. Now let us look into this issue for microwave transmission lines. Figure 1(a) depicts a generalized model of a curved metallic transmission line printed on dielectric substrate. We assume that the straight transmission line supports propagating modes in which the EM field along the tangential direction (normal to the propagating direction) is equiphase. The field energy, on the other hand, decays quickly but remains finite in the transverse aperture.

At the bend when the propagating direction keeps changing, the wavefront needs to be maintained so as to avoid the radiation loss and mode conversion. In other words, the phase velocity *v*_*p*_ should be identical as


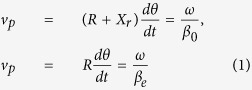


in which *β*_*0*_ is the phase constant in the air and *β*_*e*_ is that of the guided wave with correspondence of the effective permittivity *ε*_*eff*_ of the transmission line. *R* is the bending radius and *X*_*r*_ is the transverse distance where the refraction of the energy in the guided mode is lost to radiation. A relation is derived from equation (1) that


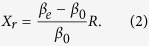


Equation (2) indicates two important properties. First, the fraction of electromagnetic wave at *X* > *X*_*r*_ doesn’t propagate fast enough to catch the equiphase fronts and hence is lost to radiation. Therefore, for a specific curved open waveguide with a fixed *β*_*e*_, a smaller bending radius *R* results in more radiation loss. Second, in order to achieve high transmission coefficient through a small-radius bend, transmission lines with strong field confinements and high phase constant *β*_*e*_ should be adopted. In the following sections, we will exam the SPP waveguide from these points of view.

### SPP dispersions of the plasmonic waveguide

**Figure 2:**
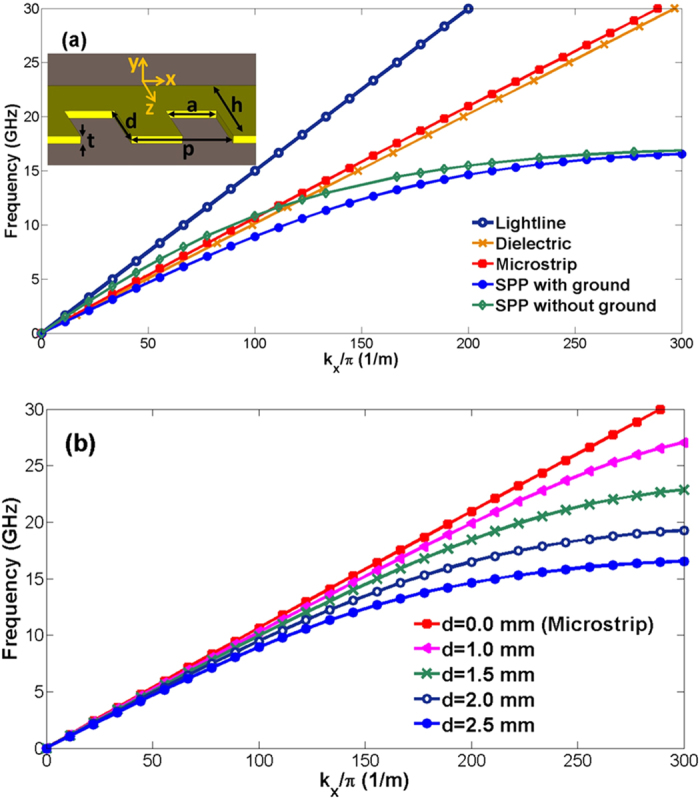
(**a**) Dispersion diagrams for different kinds of waveguides. Here, *p* = 3 mm, *a* = 1.5 mm, *d* = 2.5 mm, *h* = 5 mm and *t* = 0.07 mm. The substrate is Rogers RT5880 with the thickness of 1.575 mm, relative permittivity εr = 2.2 and loss tangent 0.0009. The coordinate in the inset is applied throughout this paper. (**b**) Simulated dispersion diagrams of spoof SPP waveguides with different groove depths *d*.

At microwave frequency, corrugated metallic structures are printed periodically on supporting dielectric substrate to propagate spoof SPPs. For a decent comparison between the SPP TL and the microstrip line, we choose grounded one-side single-strip SPP structure in this paper. Geometric parameters of the spoof SPP structure are denoted in the inset of Fig. 2(a), including the groove depth *d*, groove width *a*, strip thickness *t*, and period *p*. Such plasmonic waveguide supports surface mode with


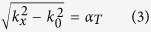


where *k*_*x*_ is the wave number along the propagation direction (*x-* direction here). *k*_*0*_ = ω/c is the wave number in air region, and *α*_*T*_ represents the decay constant along the tangential direction (*y-* and *z-* direction here). From equation (3) we conclude that when the wave number *k*_*x*_ increases, α_T_ becomes larger, and the EM field decays faster. In other words, the larger *k*_*x*_ results in the stronger field confinement.

Figure 2(a) compares the dispersion characteristic, which includes most propagating information of the guided wave, for different types of waveguide. Geometric parameters are carefully chosen as *p* = 3 mm, *a* = 1.5 mm, *d* = 2.5 mm, *h* = 5 mm and *t* = 0.07 mm. The substrate is Rogers RT5880 with the thickness of 1.575 mm, relative permittivity ε_r_ = 2.2 and loss tangent 0.0009. Note that we choose a thick substrate because it has high power capacity. For the microstrip line, the dispersion curve (the dotted red line) locates in between the light line and that for the dielectric waveguide. The dispersion curve for the ungrounded single-strip SPP TL (the green line with squares) intersects with that for the microstrip line at a specific frequency of *f*_*int*_. Below *f*_*int*_, the SPP TL possesses smaller wave number *k*_*x*_, weaker field confinement and lower loss in substrate, and presents higher transmission efficiency when compared with the microstrip line. Above *f*_*int*_, the SPP TL possesses larger wave number *k*_*x*_, stronger field confinement, and higher loss, as has been analyzed in literature^12^. Differently, the dispersion curve for the grounded single-strip SPP TL (the blue line with dots) is always on the right of that for the microstrip line. Therefore, we conclude that, below the cut-off frequency *f*_*cut-off*_, which is around 16.5 GHz as is shown in Fig. 2(a), the grounded SPP TL obtains stronger field confinements and higher phase constant *β*_*e*_(the real part of *k*_*x*_), and hence is able to achieve higher transmission coefficient through a small-radius bend, as is indicated by equation (2). Here, to be noted, when compared to the real part of *k*_*x*_, the imaginary part, which represents the metal and dielectric loss, could be ignored^13^.

We remark that the structure period *p* is in sub-wavelength scale so as to deeply confine the electromagnetic wave. It has been analyzed that the groove depth *d* has dominant impact on the wave number *k*_*x*_ of SPP TL^14^. Figure 2(b) shows the variation of dispersion curve when *d* changes from 0 (the microstrip line) to 2.5 mm. Evidently, SPP TL can be achieved from the corresponding microstrip line and controlled with flexible dispersion property.

### Transmission efficiency of curved SPP TL

Perturbation analysis has been reported in literatures on different kinds of microstrip bends and proved that curved microstrip has the highest transmission efficiency and the lowest reflection when compared with the chamfered microstrip and the right-angled one^15^. Hence, we choose the curved microstrip line as a counterpart to the curved SPP TL.

**Figure 3:**
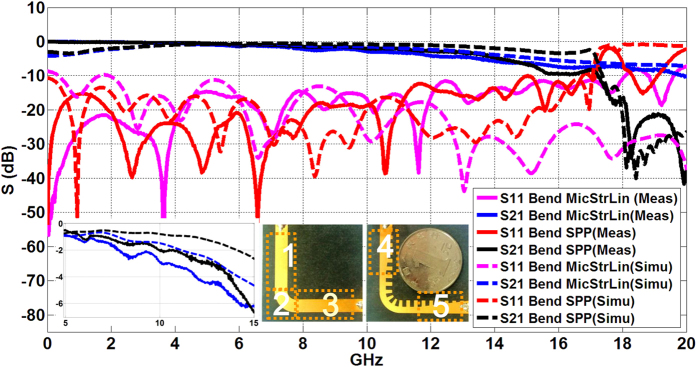
Comparison of the transmission coefficient (S21) and the reflection coefficient (S11) between the curved microstrip line and the curved SPP TL. The inset diagram in the bottom left corner is the enlarged view of S21 from 5 to 15 GHz. The inset photos are the prototypes of the curved microstrip line and SPP TL.

The left inset photo in Fig. 3 shows the 40 mm × 40 mm prototype of the curved microstrip line with bending angle *θ* = 90° and bending radius *R* = 18/π (0.19 λ_0_ at 10 GHz) in Section ‘2’. *θ* and *R* are defined in Fig. 1. In section ‘1’ and ‘3’, straight microstrip lines are connected to the input port and output port, respectively. For fair comparison, the SPP TL prototype has the same size, as is shown in the right inset photo. Geometric parameters of the SPP structure, as well as the substrate, are the same as given in the caption of Fig. 2(a). It should be pointed out that the SPP TL cannot be fed directly by the same input due to mismatching of momentum and impedance^16^,^17^. Therefore, a compact conversion section is inserted between the microstrip and the SPP TL. For the grounded one-side single-strip SPP structure, the conversion section is composed of corrugated strips with gradient grooves of height^18^, as is marked in Section ‘4’ and ‘5’.

The simulated and measured transmission coefficient (S21) and reflection coefficient (S11) are also plotted in Fig. 3. We observe that the measured transmission coefficient of the SPP TL is higher than that of the microstrip line from 5 to 14.8 GHz, and is, in particular, more than 1 dB higher from 8.4 to 14.2 GHz, as is given in the enlarged view. On the other hand, the reflection coefficient of the SPP TL is slightly lower than that of the microstrip line at most frequency points, thanks to the conversion section for impedance matching. To be noted, it has been demonstrated^9^ that the straight grounded single-strip SPP TL has higher loss than straight microstrip. Accordingly, we can conclude that the bending section in SPP TL can increase the transmission coefficient by more than 1 dB from 8.4 to 14.2 GHz. In addition, we remark that the simulated and measured cut-off frequency is about 17 GHz, which is slightly higher than the theoretical cut-off frequency *f*_*cut-off*_ observed in Fig. 2. This is due to the fact that the structures are squeezed at the bending section and hence the dispersion property is changed weakly. Other discrepancies between the simulated and measured results may come from the assembly error and inhomogeneous parameters of dielectric layer in experiments, which are not considered in the previous simulations.

**Figure 4:**
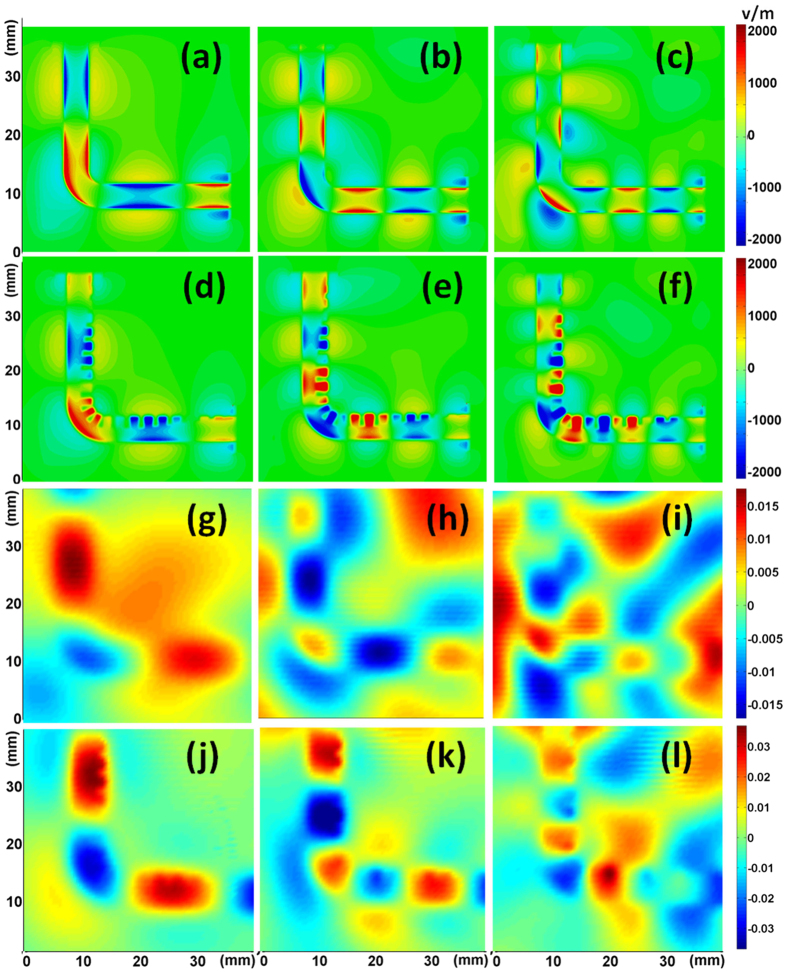
(**a**–**f**) The near-electric-field distribution (y components) that is simulated in an x-z plane 0.1 mm above the structures. (**a**–**c**) For the microstrip at 6 GHz, 9.5 GHz, and 13.5 GHz, respectively. (**d**–**f**) For the SPP TL at 6 GHz, 9.5 GHz, and 13.5 GHz, respectively. (**g**–**l**) The near-electric-field distribution that is measured in a plane 1 mm above the prototypes. (**g**–**i**) For the microstrip at 6 GHz, 9.5 GHz, and 13.5 GHz, respectively. (**j**–**l**) For the SPP TL at 6 GHz, 9.5 GHz, and 13.5 GHz, respectively.

To visualize the transmission performance, we also simulate and measure the near-field distributions (see Fig. 4). We observe from the simulated results that EM wave propagates along both TLs at 6, 9.5 and 13.5 GHz (figures (a–f)). However, for the conventional microstrip, electromagnetic field is less restricted and more fraction of electromagnetic wave is lost to radiation (see figures (a–c)). Figure 4(g–l) illustrate the measured electric fields at 6, 9.5 and 13.5 GHz. We observe significant reduction of radiation loss for the SPP TL, especially at high frequency when the size of the bending section becomes comparable to the wavelength.

### Energy loss at curvature

**Figure 5:**
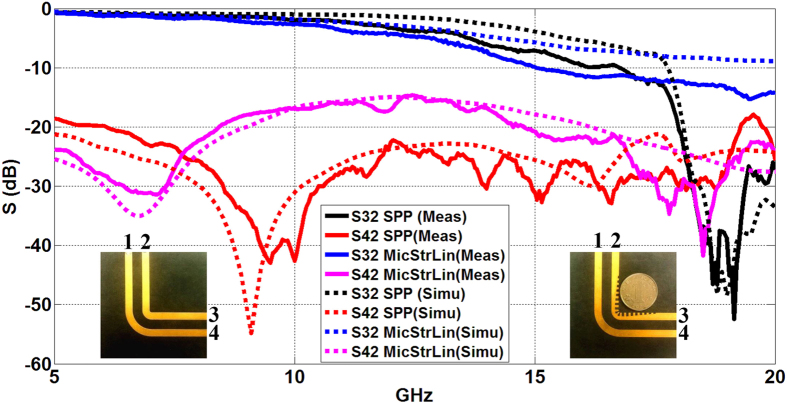
Comparison of the transmission coefficient (S32) and the coupling coefficient (S42) between the two kinds of dual-TLs. The inset photos are the prototypes of the dual curved microstrip lines (left) and the curved SPP plus microstrip TLs (right). Four ports are labeled and Port 2 is the input port. The separation between the inner and outer TL is 6.46 mm.

Next, we evaluate the crosstalk caused by radiation loss at curvature from the point of view of electromagnetic compatibility (EMC). We add a transmission line outside the microstrip line/SPP TL, as is shown in the inset photos in Fig. 5, to receive the EM wave that is radiated to the free space. The left photo shows the dual curved microstrip lines and the right one shows the curved SPP plus microstrip TLs. Ports 1–4 are labeled and Port 2 is the input port. It should be pointed out that we use microstrip to surround the curved SPP TL instead of another SPP TL. This is mainly due to two considerations. 1) The wave momentum of SPP TL is much larger than that in the air, which leads to low coupling efficiency between the radiating wave and the SPP wave. 2) Coupling between two straight microstrips and two straight SPP TLs with the same separation (6.46 mm here) are quite different^9^ and we do not want to include this difference in our comparison.

In Fig. 5, the transmission coefficient is represented by S32. Besides, a portion of energy radiates at the curvature, and is received by the surrounding microstrip. We quantify such radiation loss by S42 (the coupling coefficient). Two features are observed from the results. First, the inner SPP TL has higher transmission efficiency than the inner microstrip from 5 to 17 GHz. Second, the coupling coefficient is significantly lower for the SPP TL from 7.7 to 17 GHz, indicating that energy loss at the bending section of SPP TL is much less than that at the bending microstrip. We remarked that the coupling coefficient is higher for the SPP TL from 5 to 7.7 GHz. This may be due to the fact that at low frequencies when the wavelength is much larger than the separation, the effect of field enhancement overcomes the effect of field confinement.

**Figure 6:**
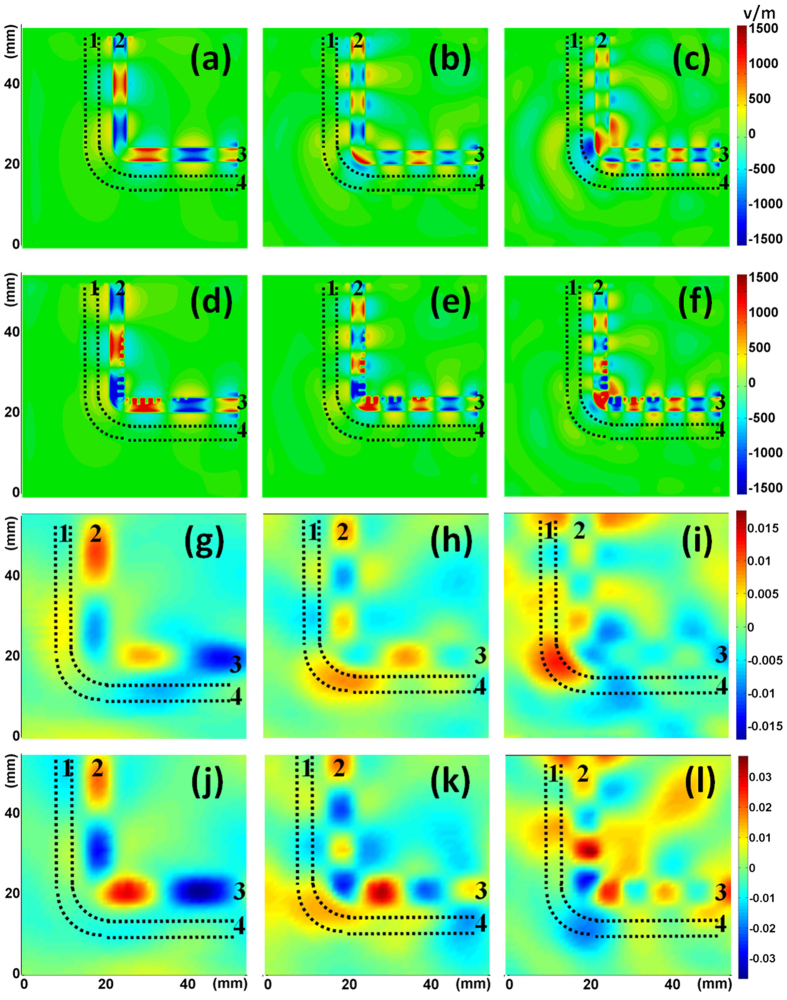
(**a**–**f**) The near-electric-field distribution (y components) that is simulated in an x-z plane 0.1 mm above the structures. (**a**–**c**) For the dual curved microstrip lines at 6 GHz, 9.8 GHz, and 12.7 GHz, respectively. (**d**–**f**) For the curved SPP plus microstrip TLs 6 GHz, 9.8 GHz, and 12.7 GHz, respectively. (**g**–**l**) The near-electric-field distribution that is measured in a plane 1 mm above the prototypes. (**g**–**i**) For the dual curved microstrip lines at 6 GHz, 9.8 GHz, and 12.7 GHz, respectively. (**j**–**l**) For the curved SPP plus microstrip TLs at 6 GHz, 9.8 GHz, and 12.7 GHz, respectively.

The visualized near-field distribution of the two kinds of dual-TL is simulated at 6, 9.8 and 12.7 GHz (see Fig. 6(a–f)). Two features are observed. First, coupling between the inner and the outer TL is pretty low at the straight sections for both dual-TLs. Therefore, we suppose that most energy received at Port 4 comes from the bending section. Second, stronger radiation happens at the bending section for the dual curved microstrip lines, especially at high frequency when the size of the bending section becomes comparable to the wavelength. The near-field distributions are also demonstrated in experiment at the same frequency points (see Fig. 6(g–l)). Figures (j–l) shows that the EM field is better confined to the inner TL and less energy radiates to the environment when the SPP TL is adopted. Therefore, EM wave can be more efficiently guided through the curved SPP TL. In contrast, nonnegligible electric field is observed “escape” from the inner microstrip and couple to the outer microstrip or radiate to the environment (see Fig. 6(g–i)). As a result, the field distributions in figures (g–i) are messier than those in (j–l). In other words, EM wave cannot be efficiently guided through the curved microstrip.

## Conclusions

The SPP TL has been demonstrated from theory and in experiment to surpass the conventional microstrip line in suppressing radiation loss at small-radius bend. This merit is resulted from the unique property that the SPP TL propagates EM wave with strong field confinements and high phase constant. In addition, the SPP TL can obtain flexible EM property through controllable design of the metallic grooves. In view of this, we expect to achieve more compact and more reliable circuits by integrating SPP TLs in existing microstrip circuits, and adopt such planar plasmonic waveguide in modern large-scale high-integrated communication systems in the future.

## Methods

Numerical simulations were performed by the commercial software, CST Microwave Studio. The simulated near-electric-field distributions (y components) were plotted in an x-z plane 0.1 mm above the TLs. To fabricate the prototypes, metallic strips were printed on Rogers RT5880 with the thickness of 1.575 mm, relative permittivity εr = 2.2 and loss tangent 0.0009. In experiment, we employed Agilent vector network analyzer (VNA) N5230C to measure the S parameters. The plot of near-field distribution was carried out in our near-field mapping system including a Vector Network Analyzer (Agilent N5230C), a metallic platform controlled by a stepper motor, and a detecting probe above the sample. To mimic the open space, we cover the metallic platform using cystosepiment with the thickness of 5 cm. It should be pointed out that the measured electric field in the 0.1 mm plane (which is used in the simulation) will be disturbed seriously due to the roughness of cystosepiment surface. Therefore, in order to guarantee the correctness of the measured near-field distribution, we perform the measurement in the 1 mm plane.

## Additional Information

**How to cite this article**: Tang, W. X. *et al*. Reduction of radiation loss at small-radius bend using spoof surface plasmon polariton transmission line. *Sci. Rep.* **7**, 41077; doi: 10.1038/srep41077 (2017).

**Publisher's note:** Springer Nature remains neutral with regard to jurisdictional claims in published maps and institutional affiliations.
